# Analysis and forecast of the disease burden of trachoma in China and the Global Population over 15 years of age, 1990–2021

**DOI:** 10.1371/journal.pntd.0013155

**Published:** 2025-05-27

**Authors:** Chaohui Li, Hui Liu, Kai Wang

**Affiliations:** 1 School of Public Health, Xinjiang Medical University, Urumqi, Xinjiang, China; 2 College of Medical Engineering and Technology, Xinjiang Medical University, Urumqi, Xinjiang, China; 3 Institute of Medical Engineering Interdisciplinary Research, Urumqi, Xinjiang, China; Yale University School of Medicine, UNITED STATES OF AMERICA

## Abstract

**Objectives:**

Trachoma is primarily transmitted through direct contact, and its complications—such as trichiasis and corneal opacity—significantly impair patients’ quality of life and result in substantial productivity losses. This study explores the differences in disability-adjusted life years (DALYs) and prevalence of trachoma globally, across regions with varying Socio-demographic Index (SDI) levels, and in China, while also projecting future trends specific to China.

**Method:**

This study, based on data from the Global Burden of Disease (GBD) database, utilized Joinpoint regression to analyze temporal trends in the age-standardized prevalence rate (ASPR) of trachoma in China from 1990 to 2021. The age-period-cohort (APC) model is used to estimate the net effects of age, period, and cohort on disease burden. Through decomposition analysis, the impact of aging, population growth, and epidemiological changes on trachoma disease burden is explored. In addition, the Bayesian Age-Period-Cohort (BAPC) model was used to project trends in the disability-adjusted life years (DALYs) rate and prevalence over the next 15 years, offering valuable insights for optimizing prevention and control strategies and consolidating achievements in disease elimination.

**Results:**

From 1990 to 2021, the ASPR and ASDR of trachoma exhibited a consistent downward trend in China, globally, and across regions with varying SDI levels. In 2021, the crude prevalence and crude DALYs rates of trachoma in China increased with age, reaching their peak in the 70–74-year age group. Marked disparities were observed among different SDI regions, with high-SDI areas recording the lowest prevalence and DALYs burden, while low-SDI regions experienced the highest. It is expected that from 2022 to 2036, the ASPR and ASDR of male and female populations aged 15 and above in China will both show a downward trend and continue to approach 0.

**Conclusion:**

The continuous decline in the burden of trachoma disease in China from 1990 to 2021 indicates significant achievements in trachoma prevention and treatment. However, attention still needs to be paid to the elderly population aged 60 and above, and health education for the entire population should be strengthened to reduce the disease burden caused by trachoma in China.

## Author summary

By analyzing the prevalence and DALYs associated with trachoma across China, the global population, and five distinct SDI regions, this study found a marked decline in both prevalence and DALYs over time. These findings suggest that the implementation of the World Health Organization’s recommended SAFE strategy has achieved substantial progress toward the elimination of trachoma. The rate of decline in China was notably faster than in other regions, with middle- and low-SDI regions also experiencing more pronounced reductions. In addition, In addition, a comparison was made between the gender, age group, and the number of cases and DALYs of trachoma patients in China and global. It was found that the number of trachoma cases and DALYs in China increased with age, and the number of cases and DALYs in women of all age groups during the same period was higher than that in men. Although the prevalence of trachoma in China is continuously decreasing, as an infectious eye disease, there are still sporadic cases of trachoma, and small-scale outbreaks may occur again in areas with poor sanitation and economic backwardness. It is still necessary to continue to popularize trachoma prevention and control knowledge and prevent the recurrence of trachoma outbreaks.

## Introduction

Trachoma is a severe blinding ocular disease caused by *Chlamydia trachomatis* (CT), an obligate intracellular bacterium [1–3]. CT is a Gram-negative, prokaryotic microorganism that strictly parasitizes eukaryotic cells and exhibits a unique biphasic developmental cycle. It infects the epithelial layer of mucosal surfaces in humans. Repeated infections with CT can lead to significant scarring on the inner surface of the eyelid, resulting in trichiasis, where the eyelashes turn inward and rub against the eyeball, causing persistent pain and photophobia [4,5]. The blindness caused by trachoma is irreversible. Trachoma is often mainly concentrated in the poorest countries, where they live in hot and dry climates with limited access to adequate water supply, weak sanitation conditions, and scarce medical resources [1,3,6–8].

According to the WHO report in 2024, approximately 103 million people live in trachoma-endemic areas and are at risk of trachomatous blindness [9]. China, as a large country with a large population, has adopted the SAFE strategy (Surgery, Antibiotics, Facial Cleansing and Environmental Enhancement). However, due to regional disparities in development and the mobility of the population, potential risks remain and warrant continued vigilance [1,10–12]. By comparing data from China, the global landscape, and regions with varying Socio-demographic Index (SDI) levels, this study aims to elucidate the synergistic effects of healthcare resource allocation, economic development, and disease control policies in the fight against trachoma.

## Methods

### Ethics statement

The data analyzed in this study were obtained from the Global Burden of Disease Study database, are secondary data, do not contain personally identifiable information, and therefore do not require ethical approval.

### Data collection

The research data is from the 2021 GBD public Database((https://vizhub.healthdata.org/gbd-results/). GBD provides epidemiological data and disease burden assessment results for 371 diseases and injuries in 204 countries and regions worldwide [13]. The data collection of this database uses spatiotemporal regression models and Bayesian prediction methods to calculate data and determine 95% uncertainty intervals (UI), enhancing the consistency of epidemiological parameters [14]. Data related to trachoma were retrieved via the Global Health Data Exchange (GHDx) platform and include prevalence and DALYs data for China and the global population from 1990 to 2021. These data serve as key indicators for assessing the burden of trachoma.

Using the GBD Results Tool, the disease name “Trachoma” was selected, along with regions “China,” “Global,” and five distinct SDI regions; the time frame was set to “1990–2021.” The metrics “number” and “rate” were chosen, and the analytical indicators included DALYs and Prevalence. For sex, the options “both,” “male,” and “female” were selected. For age, the range “15–19 years” to “95+ years” was selected, along with “age-standardized”(with five-year age groups as the standard). Finally, the data were downloaded.

### Statistic analysis

#### Descriptive analysis.

Describe the burden of trachoma disease in China, the world, and different SDI regions, analyze the disease burden in various regions around the world, and conduct in-depth analysis of the trachoma disease burden in China and global based on different age groups and genders.

#### Joinpoint regression analysis.

To quantify the observed trend changes, Joinpoint (version 5.1.0.0) was used to analyze the temporal changes in the distribution trend of trachoma in Chinese ASPR from 1990 to 2021. In addition, the segmented regression method using Joinpoint software [15]. Visualized data from different time points. The maximum number of connection points is set to 5. Calculate the Average Annual Percent Change (AAPC) and its 95% confidence interval (CI). If AAPC is greater than 0, it indicates an upward trend; if it is less than 0, it indicates a downward trend. The significance test uses Monte Carlo permutation method, and P < 0.05 indicates statistical significance.

#### Age-period-cohort model.

The age-period-cohort (APC) model based on Poisson distribution can simultaneously estimate the net effect of age, period, and cohort on disease burden [16]. This article uses a web analysis tool developed by the National Cancer Institute in the United States for age-period-cohort analysis. The APC model requires equal age, period, and time interval of the cohort, so the period is divided into a series of 5-year time periods from 1992-1996 to 2017–2021 (data from 1990 and 1991 are not included in the analysis as they are less than 5 years). The output results include local drift values, longitudinal age curves, and period (cohort) rate ratios (RR), as well as local drift values [17–19]. Representing the annual percentage change in disease rates for different age groups, the longitudinal age curve represents the age group disease rates adjusted for period bias, RR represents the relative risk of the period (cohort), RR value>1 indicates higher relative risk, and RR value<1 indicates lower relative risk.

#### Decomposition analysis model.

The model for analysis is based on the decomposition method proposed by Das Gupta [20,21],by using mathematical decomposition techniques, the overall changes are decomposed into multiple parts to identify the specific contributions of each part. Here, we explore the impact of three factors: aging, population growth, and epidemiological changes on the burden of trachoma disease.

#### Bayesian age-period-cohort models.

The Bayesian Age-Period-Cohort (BAPC) model approximates posterior marginal distributions [22] using integrated nested Laplace approximations (INLA). To predict the trend of trachoma prevalence and DALYs rate in China, BAPC analysis was performed using the R-BAPC package and the R-INLA package to predict the standardized prevalence of trachoma and the rate of standardized DALYs by gender in China from 2022 to 2036. In this paper, we use Excel 2021 software to organize and analyze the data of China, the world, and the high SDI region from 1990 to 2021, and the data analysis and visualization are performed by R (version 4.4.2).

## Results

### Epidemiologic analysis of trachoma in China and global and in five SDI regions, 1990 and 2021

Table 1 illustrates that in 1990, the low SDI region reported the highest number of trachoma cases, with a prevalence of 1,169,228, followed by the low-middle SDI region with 597,522 cases. The middle SDI region recorded 233,180 cases, while the high SDI region had the lowest prevalence, with only 44 cases. In 2021, the regional distribution of trachoma prevalence remained consistent with that of 1990; however, prevalence rates had declined across all regions. In terms of disease burden, the low SDI region also recorded the highest number of DALYs lost to trachoma in 1990, totaling 97,353 person-years, followed by the low-middle SDI region (62,402 person-years) and the middle SDI region (26,050 person-years). The high SDI region experienced the lowest burden, with only 5 person-years lost. In 2021, the regions with the greatest DALYs losses remained the same as in 1990. However, all regions showed a reduction in DALYs lost to trachoma over time.

**Table 1:** Prevalence of trachoma and DALYs in China and global and in five SDI regions in 1990 and 2021.

			Global (95% UI)	China (95% UI)	High SDI (95% UI)	High-middle SDl (95% UI)	Middle SDl (95% UI)	Low-middle SDl (95% UI)	Low SDI (95% UI)
1990	Prevalence	number of cases	2026157 (1630673,2521481)	48544 (35888,64870)	44 (29,63)	25064 (20380,30682)	233180 (175668,300247)	597522 (455614,759348)	1169228 (962845,1422202)
		Age-Standardized rate (per 100,000)	51 (41,63)	6 (5,9)	0 (0,0)	3 (2,3)	26 (19,33)	106 (81,136)	541 (446,650)
	DALYs	number of cases	187730 (123976,261508)	5683 (3408,9078)	5 (3,9)	1825 (1254,2470)	26050 (16348,39076)	62402 (39017,92500)	97353 (66945,133005)
		Age-Standardized rate (per 100,000)	5 (3,7)	1 (0,1)	0 (0,0)	0 (0,0)	3 (2,4)	11 (7,17)	46 (31,62)
2021	Prevalence	number of cases	1414047 (1100377,1799564)	12586 (8426,17662)	76 (45,122)	9171 (6514,12747)	156529 (116503,205520)	283478 (204430,377295)	963947 (758812,1216319)
		Age-Standardized rate (per 100,000)	16 (13,21)	1 (0,1)	0 (0,0)	0 (0,1)	6(5,8)	21 (15,28)	198 (157,250)
	DALYs	number of cases	123190 (80954,174325)	1726 (922,2836)	7 (4,11)	728 (466,1052)	15180 (9362,22537)	31950 (19240,48686)	75256 (51206,104406)
		Age-Standardized rate (per 100,000)	1 (1,2)	0 (0,0)	0 (0,0)	0 (0,0)	1 (0,1)	2 (1,4)	16 (11,21)
ASPR of AAPC(%)			-3.60	-7.39	-0.43	-5.18	-4.52	-5.02	-3.19
ASDR of AAPC(%)			-3.77	-6.86	-1.52	-4.91	-4.95	-4.80	-3.39

**Abbreviations:** DALYs, Disability adjusted life years; SDI, socio-demographic index; UI, uncertainty interval; ASPR, age-standardized prevalence rate; ASDR, age-standardized DALYs rate; AAPC, Average Annual Percent Change.

AAPC < 0 indicates that the indicator shows an average annual downward trend. AAPC > 0 indicates that the indicator shows an average annual upward trend.

### Global burden of disease by region

#### Distribution of prevalence by region of the world in 1990 and 2021.

**Fig 1:**
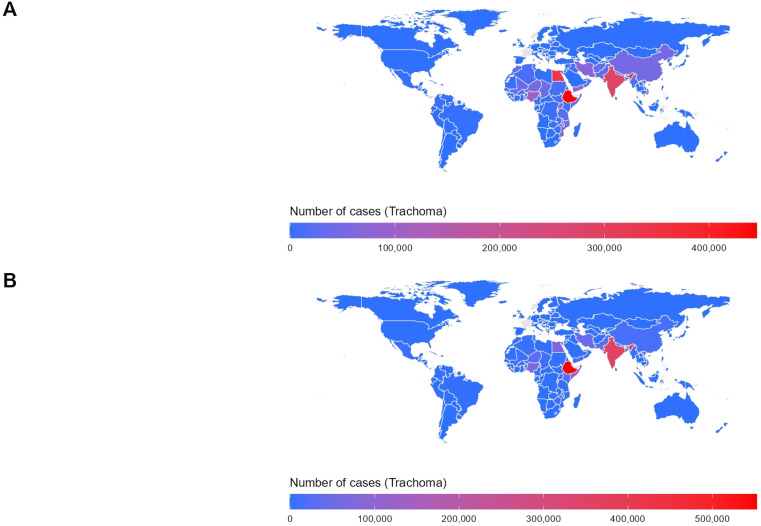
Number of trachoma cases worldwide. (A) the number of cases in various regions of the world in 1990. (B) the number of cases in various regions of the world in 2021; The shapefiles used for spatial visualization are sourced from Natural Earth. The terms of free use for these shapefiles can be found at: https://www.naturalearthdata.com/about/terms-of-use/, the shapefiles available at: https://www.naturalearthdata.com/downloads/50m-cultural-vectors/50m-admin-0-countries-2/.

As shown in Fig 1, the number of trachoma cases in 2021 was significantly lower than in 1990, both in total and across individual regions. In 1990, the Federal Democratic Republic of Ethiopia reported the highest number of cases, totaling 445,062, followed by the Arab Republic of Egypt with 341,601 cases, and the Republic of India with 281,079 cases, ranking third. In 2021, although the overall prevalence declined, the Federal Democratic Republic of Ethiopia still reported the highest number of cases, increasing to 552,128. It was followed by the Republic of India with 356,503 cases, and the Federal Republic of Somalia, which ranked third with 103,882 cases.

### Distribution of DALYs in various regions of the World in 1990 and 2021

**Fig 2:**
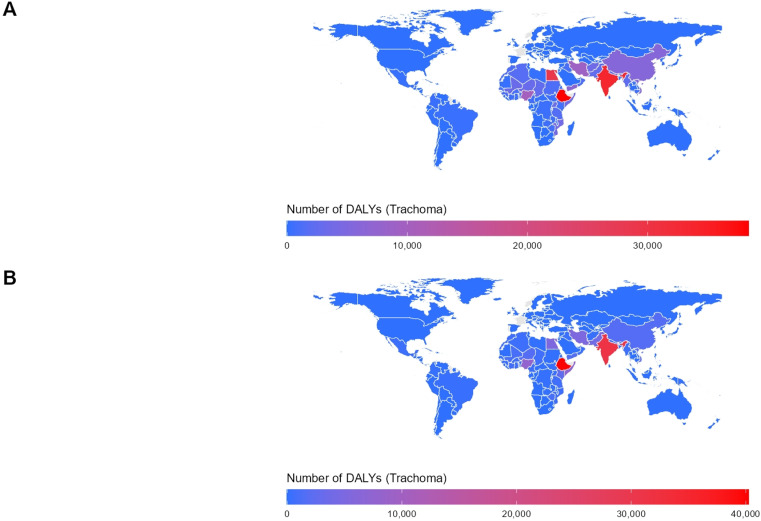
Number of DALYs in trachoma worldwide. (A) the DALYs in various regions of the world in 1990, (B) the DALYs in various regions of the world in 2021. The shapefiles used for spatial visualization are sourced from Natural Earth. The terms of free use for these shapefiles can be found at: https://www.naturalearthdata.com/about/terms-of-use/, the shapefiles available at: https://www.naturalearthdata.com/downloads/50m-cultural-vectors/50m-admin-0-countries-2/.

As shown in Fig 2, the number of DALYs lost due to trachoma in 2021 was significantly lower than in 1990, both in total and across all regions. In 1990, the Federal Democratic Republic of Ethiopia reported the highest DALYs burden, with 38,374 person-years lost, followed by the Republic of India with 33,588 person-years, and the Arab Republic of Egypt, which ranked third with 28,875 person-years lost due to trachoma. In 2021, although the overall burden had decreased, the Federal Democratic Republic of Ethiopia continued to experience the highest number of DALYs lost, with 40,209 person-years, followed by the Republic of India with 31,432 person-years. The Federal Republic of Nigeria ranked third, with 7,844 person-years of DALYs lost due to trachoma.

### Joinpoint regression model for the burden of trachoma disease in China from 1990 to 2021

**Fig 3:**
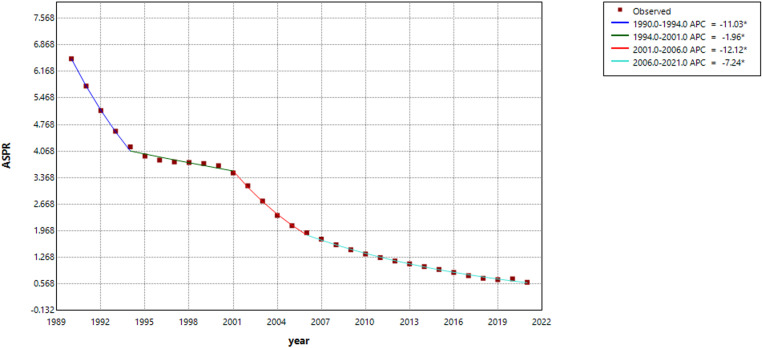
Joinpoint regression analysis of ASPR for trachoma in China.

The Joinpoint regression analysis (Fig 3) of the ASPR for trachoma in China from 1990 to 2021 illustrates notable shifts in the disease burden across different time periods. From 1990 to 1994, the ASPR exhibited a significant and sharp decline, with an Annual Percentage Change (APC) of -11.03 (t = -14.12, P < 0.001). From 1994 to 2001, the downward trend continued at a more moderate rate, with an APC of -1.96 (t = -3.95, P < 0.001).From 2001 to 2006, there was another marked decline, with an APC of -12.12 (t = -12.55, P < 0.001).Finally, during the period 2006 to 2021, the rate of decline slowed, but remained significant, with an APC of -7.24 (t = -54.73, P < 0.001).These trends reflect substantial progress in the reduction of trachoma prevalence in China over the past three decades, albeit with varying rates of decline.

### The burden of trachoma disease among different age groups in China and the world in 1990 and 2021

#### The burden of trachoma disease in various age groups in China in 1990 and 2021.

**Fig 4:**
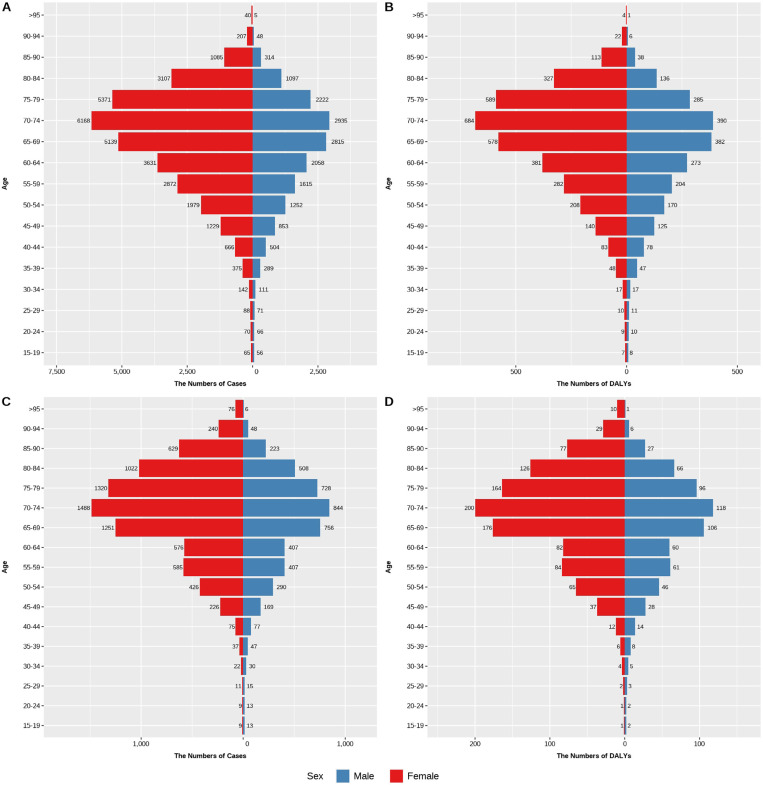
Prevalence and DALYs of various age groups in China from 1990 to 2021. (A) the number of cases in China in 1990 (B) the number of DALYs in China in 1990, (C) the number of cases in China in 2021, (D) the number of DALYs in China in 2021.

From Fig 4, it can be seen that the prevalence of trachoma and DALYs in various age groups in China in 1990 and 2021 were higher in females than in males. The number of DALYs and the number of cases reached their maximum values between the ages of 70–74 for both males and females. In 1990, Chinese males and females aged 70–74 had 2,935 and 6,168 cases respectively, with corresponding DALYs of 390 and 684 person-years. In 2021, the number of cases in this age group dropped to 844 for males and 1,488 for females, while DALYs were 118 and 200 person-years, respectively. Additionally, both the number of cases and DALYs increased with age, reaching their maximum at ages 70–74, after which they gradually declined with advancing age.

#### The burden of trachoma disease in various age groups in the World in 1990 and 2021.

**Fig 5:**
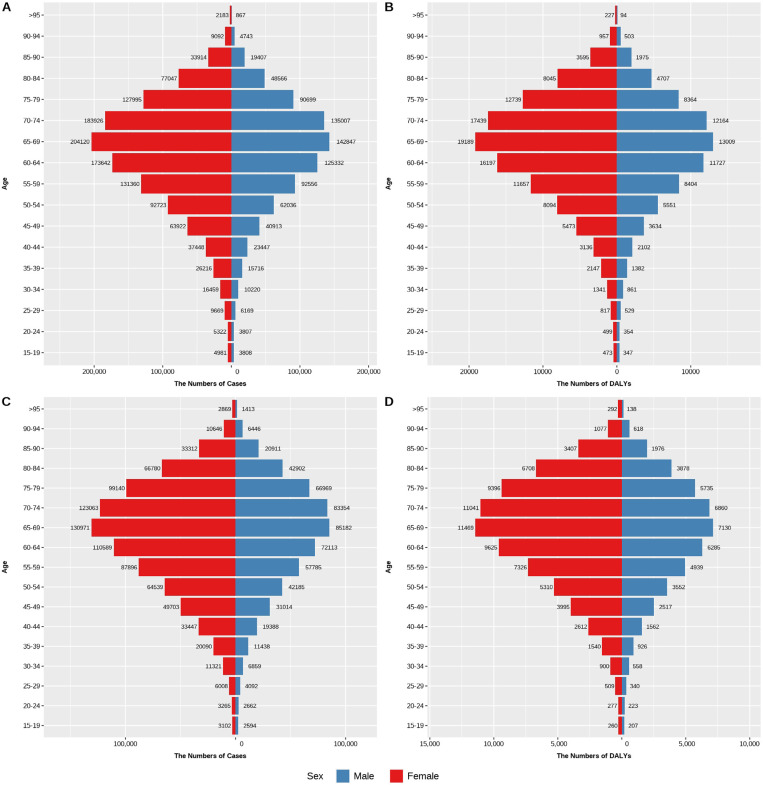
Global prevalence and DALYs in 1990 and 2021. (A) the global prevalence in 1990, (B) the global DALYs in 1990, (C) the global prevalence in 2021, (D) the global DALYs in 2021.

From Fig 5A and 5C, the number of female trachoma cases worldwide was higher than that of males in both 1990 and 2021, and the number of cases gradually increased with age, peaking in the 65–69 age group—reaching 204,120 cases in 1990 and 13,091 cases in 2021—before declining with further aging. Fig 5B and 5D indicate that global DALYs also initially increased with age in both 1990 and 2021, peaking at 65–69 years before gradually decreasing. In 1990, DALYs reached 19,189 person-years, and in 2021, they declined to 11,469 person-years. Notably, DALYs were consistently higher among females than males.

#### The disease burden between different genders in China and globally from 1990 to 2021.

**Fig 6:**
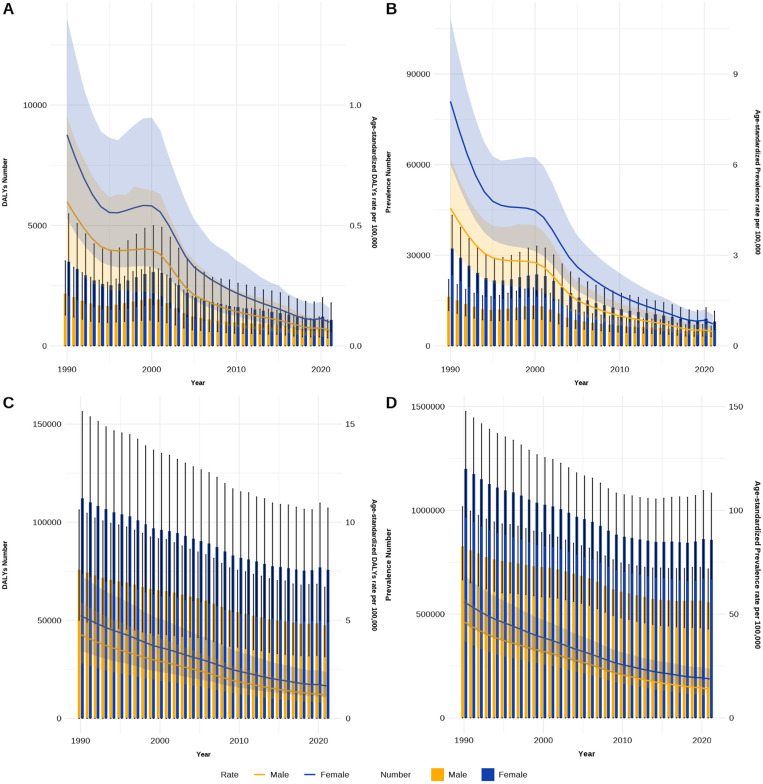
Burden of trachoma and DALYs in China and globally from 1990 to 2021. (A) the DALYs and ASDR in China,(B) the prevalence and ASPR in China, (C) the DALYs and ASDR globally, (D) the prevalence and ASPR globally.

From Fig 6, the number of trachoma cases in China decreased from 48,544 in 1990 to 12,586 in 2021, a decrease of 74.07%. Globally, the number of cases fell from 2.03 million to 1.41 million, representing a 30.21% decline. The ASPR of trachoma in China decreased from 6.50 per 100,000 in 1990 to 0.61 per 100,000 in 2021, which is significantly lower than the global ASPR (51.00 per 100,000 in 1990 and 16.37 per 100,000 in 2021).In terms of disease burden, the DALYs for trachoma in China declined from 5,683 person-years in 1990 to 1,726 person-years in 2021, a decrease of 69.63%. Globally, DALYs decreased from 187,700 person-years to 123,200 person-years, a decrease of 34.36%. The ASDR of trachoma in China has decreased from 0.75 per 100,000 person-years in 1990 to 0.08 per 100,000 in 2021, again lower than the global rates (4.76 per 100,000 in 1990 and 1.43 per 100,000 in 2021).Between 1990 and 2021, both in China and globally, the number of trachoma cases and DALYs were consistently higher in females than in males. Moreover, the age-standardized prevalence rate and age-standardized DALYs rate for trachoma showed a steady decline over time in both China and worldwide. The AAPCs of ASPR and ASDR in China are -7.39% and -6.86%, respectively, while the AAPCs of ASPR and ASDR globally are -3.60% and -3.77%, respectively (all P < 0.001; as shown in Table 1).

### The disease burden between China and the world in different age groups and genders in 1990 and 2021

**Fig 7:**
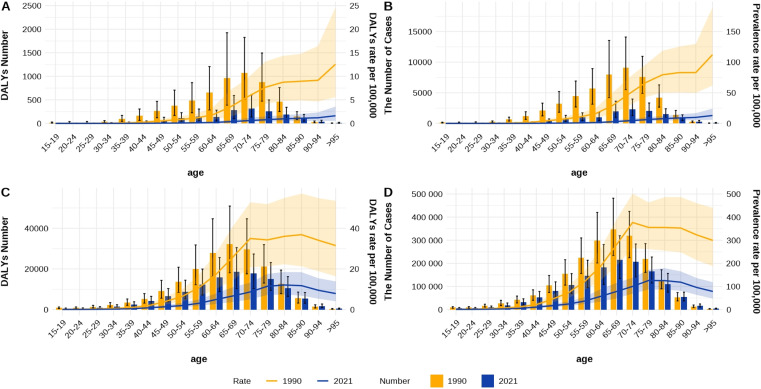
DALYs and prevalence rates of various age groups in China and globally in 1990 and 2021. (A) the China DALYs number and DALYs rate in 1990 and 2021, (B) the China prevalence and prevalence rate in 1990 and 2021. (C) the global DALYs number and DALYs rate in 1990 and 2021, (D) the global prevalence and prevalence rate in 1990 and 2021.

As shown in Fig 7A and 7B, it can be seen that the number of trachoma cases and DALYs across all age groups in China were higher in 1990 than in 2021. Both the overall prevalence and DALYs rates were significantly higher in 1990, and they gradually increased with age, reaching their maximum values above 95 years old (prevalence rate was 112.12 per 100,000 in 1990 and 129.2 per 100,000 in 2021. DALYs rate was 125.6 per 100,000 in 1990 and 1.63 per 100,000 in 2021).Fig 7C and 7D demonstrate similar trends on a global scale. In 1990, trachoma prevalence and DALYs rates across all age groups were higher than those in 2021. The overall global prevalence and DALYs rates were also higher in 1990. As age increased, the prevalence and DALYs rate gradually increased, reaching their peak between the ages of 70–74 (prevalence rate was 376.72 per 100,000 in 1990 and 100.28 per 100,000 in 2021. DALYs rate was 34.97 per 100,000 in 1990 and 8.70 per 100,000 in 2021), and then gradually stabilized. It can be seen that there are differences in the global prevalence and DALYs between 1990 and 2021.

#### The burden of disease across different SDI regions.

**Fig 8:**
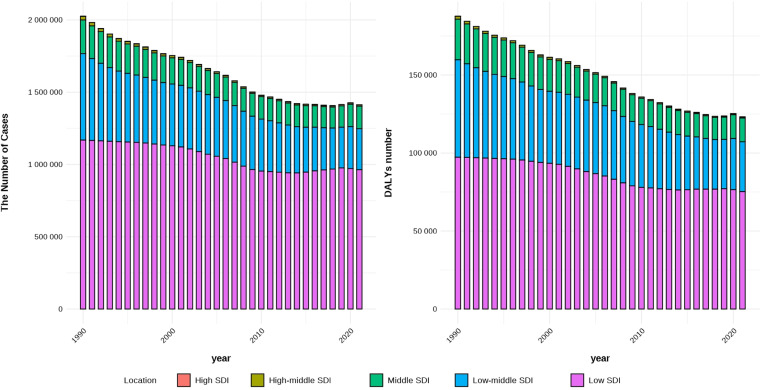
The prevalence of trachoma and DALYs among different SDI regions. (A) the number of cases in 5 SDI regions, (B) the number of DALYs in 5 SDI regions.

As shown in Fig 8, from 1990 to 2021, the number of trachoma cases declined across all regions. The number of cases was lowest in high-SDI regions among the five SDI categories, with 44 cases in 1990 and 76 in 2021. Regions with high-middle SDI followed. In contrast, the highest number of trachoma cases was observed in low-SDI regions, with 1,169,228 cases in 1990 and 963,947 in 2021.

Similarly, the highest number of disability-adjusted life years (DALYs) due to trachoma was recorded in low-SDI regions (97353 person-years in 1990 and 75256 person-years in 2021),while high-SDI regions had the lowest DALYs burden (5 person-years in 1990 and 7 person-years in 2021). It can be seen that the level of SDI is related to the prevalence and disease burden of trachoma.

### APC model analysis of trachoma patients in China from 1990 to 2021

#### APC model analysis of the prevalence of trachoma in China from 1990 to 2021.

Based on the APC model analysis of trachoma prevalence in China, the prevalence rate of trachoma among individuals aged 15 and above showed a significant downward trend from 1990 to 2021. The net drift was -7.02% (P < 0.05), indicating that, after adjusting for cohort effects, the average annual decline in the trachoma prevalence rate in this population was 7.02% during this period.

**Fig 9:**
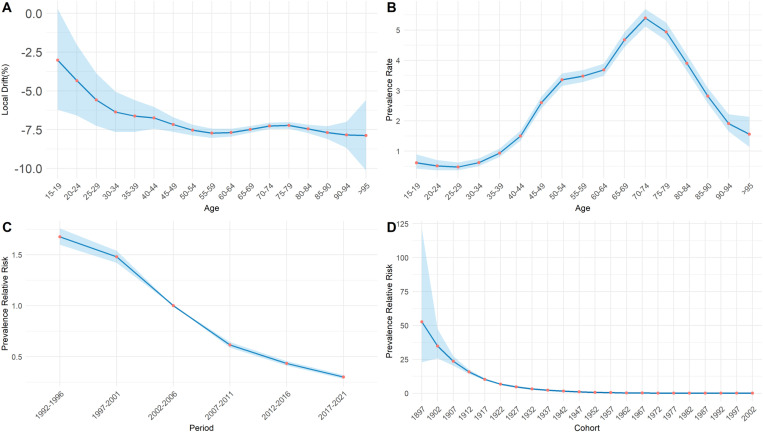
Local drift, age, period, and cohort effects of trachoma prevalence in China from 1990 to 2021. (A) the local drift in different age groups, (B) the age effect of trachoma risk, (C) the period effect of trachoma risk, (D) the cohort effect of trachoma risk.

By analyzing Fig 9A, it can be seen that the local drift values of each age group are all less than 0, and overall, the disease burden of the prevalence rate continues to decrease with age. In Fig 9B, an analysis of the population aged 15 and above in China shows that the risk of trachoma first decreases in the 15–19 age group, gradually increases in the 25–29 age group, reaches its peak in the 70–74 age group, and then gradually decreases again. The period effect presented in Fig 9C, using 2002–2006 as the reference period (RR = 1), showed an overall downward trend in trachoma risk. The lowest risk was observed during 2017–2021. Compared to 2002–2006, the risk of trachoma in 1992–1996 was 1.68 times higher (95% CI: 1.60–1.76), while in 2017–2021 it was 0.30 times lower (95% CI: 0.28–0.32).The cohort effect showed that the risk of trachoma in the early-born cohort was higher than in 1947 (RR = 1). As shown in Fig 9D, the risk steadily declined across successive birth cohorts, reaching its lowest level in the 2002 birth cohort. Specifically, the trachoma risk in the 1897 birth cohort was 52.62 times that of the 1947 cohort (95% CI: 22.89–120.97), whereas the risk in the 2002 cohort was only 0.05 times that of the 1947 cohort (95% CI: 0.02–0.15).

#### APC model analysis of the DALYs rate of trachoma in China from 1990 to 2021.

Based on the Age-Period-Cohort model analysis of trachoma DALYs in China, the DALYs rate of trachoma among individuals aged 15 and above showed a significant downward trend from 1990 to 2021. The net drift was -6.53% (P < 0.05), indicating that, after excluding the influence of the cohort effect, the average annual decline in the trachoma DALYs rate among people aged 15 and above in China during 1990–2021 was 6.53%.

**Fig 10:**
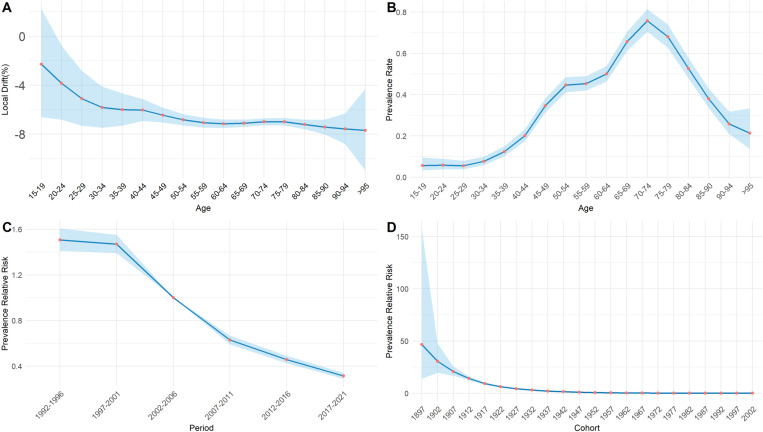
Local drift, age, period, and cohort effects of DALYs of trachoma in China from 1990 to 2021. (A) the local drift in different age groups, (B) the age effect of trachoma DALYs risk, (C) the period effect of trachoma DALYs risk, (D) the cohort effect of trachoma DALYs risk.

By analyzing Fig 10A, it can be seen that the local drift values of each age group are all less than 0, and overall, the disease burden of DALYs continues to decrease with age. In Fig 10B, an analysis of the population aged 15 and above with trachoma in China shows that the risk of DALYs first increases in the 15–19 age group, reaches its peak in the 70–74 age group, and gradually decreases. The period effect, using 2002–2006 as the reference period (RR = 1), shows an overall downward trend in DALYs risk in Fig 10C. The DALYs risk reached its lowest level in 2017–2021. Compared to 2002–2006, the DALYs risk in 1992–1996 was 1.50 times higher (95% CI: 1.41–1.61), while in 2017–2021, the DALYs risk was 0.31 times (95% CI: 0.29–0.34).The cohort effect shows that the risk of DALYs in early born cohorts is higher than in 1947 (RR = 1). Fig 10D shows that as the cohort moves forward, the risk of DALYs gradually decreases, and in 2002, the DALYs risk value reached its lowest value. The risk of DALYs in the 1897 cohort was 46.51 times higher than that in the 1947 cohort (95% CI 13.78-157.00), and the risk of DALYs in the 2002 cohort was 0.078 times higher than that in the 1947 cohort (95% CI 0.02-0.32).

### Decomposition model analysis of trachoma in China from 1990 to 2021

**Fig 11:**
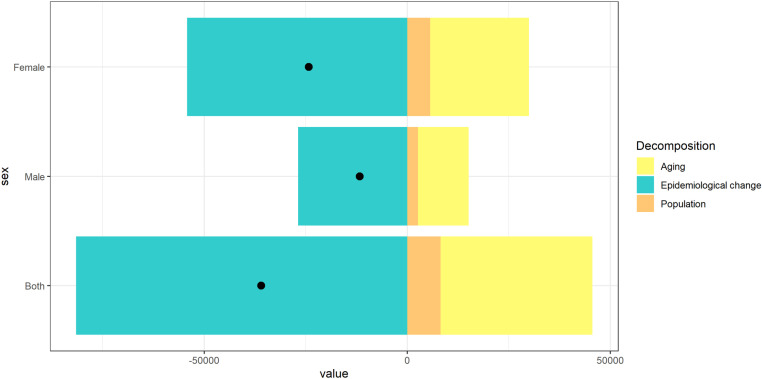
Decomposition analysis of the number of trachoma cases in China from 1990 to 2021. The changes in the burden of trachoma disease in China from 1990 to 2021 are determined by demographic factors such as population growth, aging, and epidemiological changes. The black dots represent the overall changes in the contributions of all three components. For each component, a positive value indicates a corresponding increase in the burden of trachoma disease attributed to that component; Negative values indicate a corresponding reduction in the burden of trachoma disease caused by this component.

Compared to 1990, both the number of trachoma cases and DALYs in 2021 decreased globally, in China, and across different SDI regions. Therefore, a decomposition analysis was conducted to further explore the relative contributions of three factors—population aging, population growth, and epidemiological changes—to the change in trachoma burden in China (Fig 11). The study found that the primary driver of the reduction in trachoma cases in China was epidemiological change. Among both males and females, females experienced a greater reduction in disease burden due to epidemiological shifts. Specifically, epidemiological changes and aging accounted for 223.66% and -100.41% of the total change in females, respectively (Table 2). Males accounted for 229.29% and -106.72% of the total increase, respectively, due to changes in epidemiology and aging. In addition, the increase in population and age has a significant positive effect.

**Table 2:** Decomposition analysis of trachoma prevalence in China from 1990 to 2021.

Sex	Overall difference	Aging	Population	Epidemiological change
Both	-35958.04	37436.823(-104.11%)	8187.405(-22.77%)	-81582.264(226.88%)
Male	-11728.28	12516.5(-106.72%)	2647.345(-22.57%)	-26892.125(229.29%)
Female	-24229.75	24328.698(-100.41%)	5633.859(-23.25%)	-54192.312(223.66%)

Overall difference: Total change in the burden of disease. Ageing: The impact of population ageing on the burden of disease. Population: Impact of changes in population size on the burden of disease. Epidemiologic change: Impact of changes in epidemiologic risk factors on the burden of disease. Negative values indicate a decrease in the burden of disease; positive values indicate an increase in the burden of disease.

### Projection of trachoma prevalence and DALYs rates in China from 2022 to 2036

#### Projection of trachoma prevalence in China from 2022 to 2036.

**Fig 12:**
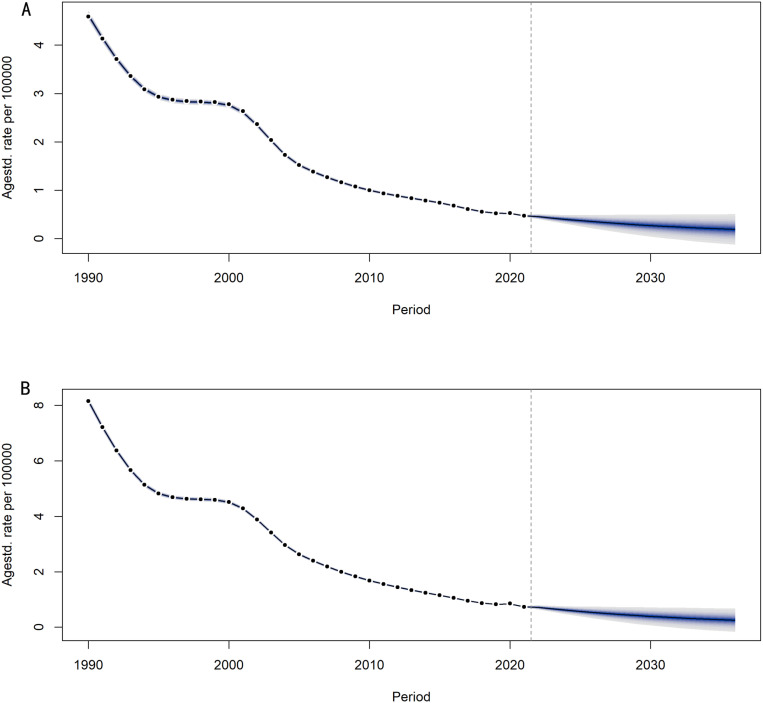
Age-standardized projections of male and female prevalence in China from 2022 to 2036. (A) the age-standardized projection of prevalence for males in China (B) the age-standardized projection of prevalence for females in China.

As shown in Fig 12, it can be seen that the downward trend of ASPR for males and females is similar. From 1990 to 1994, ASPR for males and females rapidly decreased. From 1995 to 2001, ASPR for males and females decreased slowly. From 2002 to 2021, ASPR for males and females decreased rapidly. From 2021 to 2036, it is predicted that ASPR for males and females will begin to slowly decline. The age standardized prevalence rate of male trachoma has decreased from 5 per 100,000 in 1990 to 5 per 1,000,000 in 2021, and is predicted to drop to approximately 2 per 1,000,000 by 2036. The age standardized prevalence rate of female trachoma has decreased from 8 per 100,000 in 1990 to 7 per 1,000,000 in 2021, and is predicted to approach 3 per 1,000,000 by 2036. Overall, the prevalence rates of male and female trachoma in China have shown a downward trend, indicating significant effectiveness of global trachoma prevention and control measures in the past 30 years.

#### Projection of DALYs rates for trachoma in China from 2022 to 2036.

**Fig 13:**
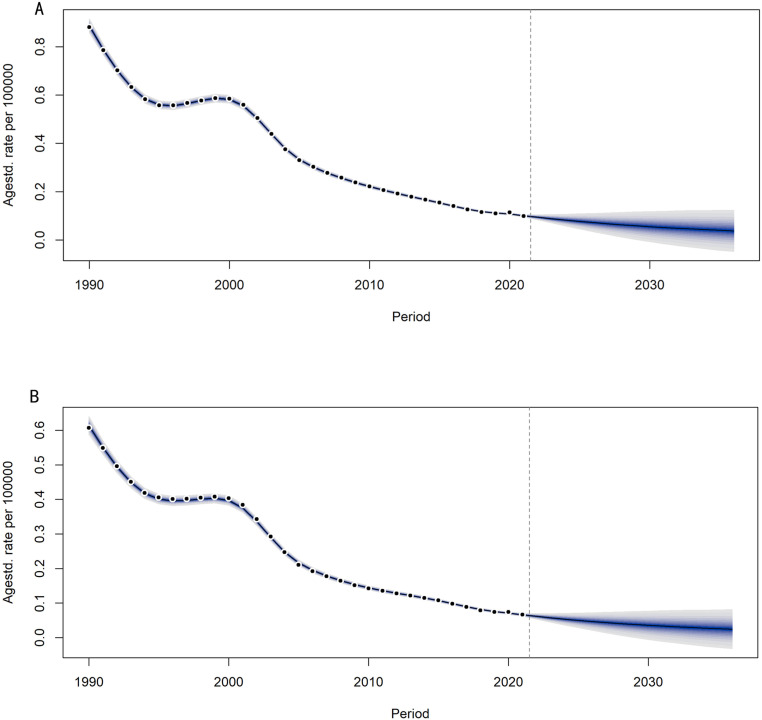
Age-Standardized Projections of DALYs Rates for Males and Females in China from 2022 to 2036. (A) the age-standardized projection of DALYs rate for Chinese females.(B) the age-standardized projection of DALYs rate for Chinese males.

From Fig 13, it can be seen that overall, the decreasing trend of ASDR in males and females is almost the same. From 1990 to 1995, ASDR in males and females rapidly decreased, and from 1995 to 2002, the decreasing rate of ASDR in trachoma slowed down. From 2003 to 2021, ASDR experienced a rapid decline, and from 2021 to 2036, the predicted decline rate is expected to slow down again. The age standardized DALYs rate for male trachoma has decreased from 6 per 1,000,000 in 1990 to 7 per 10,000,000 in 2021, and is predicted to approach 2 per 10,000,000 in 2036. The age standardized DALYs rate for female trachoma has decreased from 8 per 1,000,000 in 1990 to 1 per 1,000,000 in 2021, and is predicted to approach 4 per 10,000,000 in 2036. The predicted ASDR for males is smaller than that for females, and the predicted ASDR for both males and females is continuously approaching 0.

## Discussion

This study analyzes the trend of trachoma disease burden in China, globally, and in different SDI regions based on the Global Burden of Disease Database (GBD 2021). The research results indicate that through the SAFE strategy [23–25],Although significant progress has been made in global efforts to prevent and control trachoma, there are still high rates of trachoma and DALYs in areas with poor sanitation conditions, water scarcity, underdeveloped economic and medical resources [1,2,26–28], Related studies have shown that water supply, facial cleanliness, toilet cleanliness, health education for trachoma, and eye secretions are all related to the prevalence of trachoma [29,30]. There are lower prevalence rates and DALYs among regions with more developed economic and medical resources [31]. During the 32 years, the overall ASPR and ASDR of trachoma in China showed a downward trend, lower than the global average, and the downward trend of AAPC was much higher than the global trend. However, compared to high SDI levels, there is still a gap between standardized prevalence and DALYs. Over the past 32 years, ASPR and ASDR of trachoma in China have shown an overall downward trend, remaining below the global average. Moreover, the decline in the average annual percentage change (AAPC) in China was significantly greater than the global trend. However, compared to regions with high SDI levels, there remains a gap between the standardized prevalence rate and DALYs.

According to the Joinpoint regression model analysis, the ASPR of trachoma in China showed a significant decrease from 1990 to 2021. However, there were notable increases during the periods 1990–1994 and 2001–2006. These increases may be attributed to the inclusion of trachoma in the National Plan for the Prevention and Treatment of Blindness since the 1990s [32], where governments at all levels clarified target responsibilities and implemented precise prevention and control through epidemiological investigations and targeted screening of key populations. At the same time, economic development and the upgrading of health infrastructure contributed to a significant reduction in the number of cases. Another reason for the decline in trachoma in China was the country’s signing of the global initiative “Vision 2020: The Right to Sight” in 1999. The strategic goal of “Vision 2020” [33,34] was to eliminate avoidable cataracts, trachoma, onchocerciasis, childhood blindness, low vision, and refractive errors worldwide by the year 2020. Eliminating blinding trachoma was one of the five goals of eliminating avoidable blindness. To achieve this goal, China has included the eradication of blinding trachoma in its national blindness prevention plan. In the APC model period effect, the risk of trachoma in China showed a decreasing trend from 1990 to 2021, consistent with the results of the Joinpoint regression analysis.

In terms of gender differences, the crude prevalence rate and crude DALY rate of trachoma in males in China and globally were lower than those in females in 2021 [35–37]. Across all age groups in China, the number of trachoma cases among males was consistently lower than that among females. This discrepancy may be partly explained by the fact that women are more frequently responsible for household chores such as laundry and cleaning. In areas lacking access to safe water sources, they may frequently come into contact with sewage-contaminated with Chlamydia trachomatis (such as washing clothes by the river or cleaning their faces with dirty water). On the other hand, in some regions, women bear the primary responsibility of taking care of children in the family, and children are carriers of Chlamydia trachomatis in a group. Through close contact with infected children increases the risk of trachoma in women. In terms of age distribution, the crude prevalence and crude DALY rate of trachoma in the elderly population in China and globally were significantly higher than those in the younger population in 2021. This may be attributed to the gradual decline of the immune system function of elderly people with age, known as “immunosenescence” which weakens their defense against pathogens and makes them more susceptible to infection with Chlamydia trachomatis. At the same time, the function of the lacrimal gland in older adults decreases, and the secretion of tears decreases, leading to dryness of the ocular surface and weakened antibacterial ability, making the elderly population more susceptible to trachoma.

This study used the BAPC model to project trends in the prevalence and DALYs rates of trachoma in China from 2022 to 2036 by gender. The projections indicate that, compared to 2021, the age-standardized prevalence rate of trachoma in 2036 will approach zero. The” Healthy China 2030 Action Plan” continues to promote surveillance and intervention for trachoma to ensure that disease control efforts are sustained and not reversed. Eliminating blinding trachoma does not equate to the complete eradication of trachoma. China has a vast territory with significant economic development and environmental sanitation differences. As a contagious eye disease, trachoma still has sporadic cases, which may lead to small-scale outbreaks in areas lacking water resources, poor sanitation conditions, and underdeveloped economic and educational levels [30]. it is still necessary to continue to popularize knowledge of trachoma prevention and control and prevent the resurgence of trachoma epidemics. We need to strengthen our attention, mainly focusing on children and the elderly population. Through the SAFE strategy provided by WHO, we aim to raise public health awareness, enhance public awareness of trachoma disease, and implement early diagnosis and treatment measures to reduce the prevalence of trachoma, minimize disability caused by the disease, improve patient quality of life, and ultimately alleviate the overall burden of trachoma on individuals, families, and society.

The WHO Roadmap for Neglected Tropical Diseases 2021–2030 [9] clearly identifies trachoma as a key target for elimination, proposing its global elimination as a public health problem through “integrated interventions, cross-sector collaboration, and health system integration.” The SAFE strategy, as the cornerstone of trachoma elimination, has played a critical role in achieving a 91% reduction in the global at-risk population for trachoma between 2002 and 2023. Although the WHO 2030 targets provide a systematic framework for trachoma elimination, their success depends on the policy implementation capacity of individual countries, the rate of technological innovation adoption, and the coherence of global health governance. Looking ahead, accelerating the elimination process will require advancing international cooperation in vaccine development and optimizing the allocation of health resources.

This study also has some limitations. Firstly, the data used in this research were derived from the GBD 2021 database, which estimates values using a combination of systems dynamics and statistical modeling approaches [29,38]. Therefore, the estimates may deviate from actual conditions. Secondly, high-income countries have richer, more systematic and accurate data, while data from low-income countries or conflict areas are often incomplete or even missing, while the lack of systematic long-term health records in some countries due to incomplete historical data backdating has led to a reliance on models to fill in the judgments of historical trends. Third, this study has methodological limitations: Due to the systematic absence of trachoma epidemiological data for the 0–14 age group in the Global Burden of Disease (GBD) database (with all regional values recorded as zero for this age group), the study population was ultimately restricted to individuals aged 15 years and older to ensure data completeness. This age stratification bias may affect the evaluation of intervention effectiveness, as mass drug administration (MDA) with antibiotics in the SAFE strategy primarily targets children aged 1–14. The data gap compromises the ability to assess the cost-effectiveness of this intervention. According to WHO guidelines, children aged 1–9 are the core surveillance group for active transmission of Chlamydia trachomatis in trachoma, and the current study design is unable to capture the dynamic characteristics of this high-risk population. In addition, In addition, the projection prediction results of the BAPC model are influenced by various future changing factors [39]; due to factors such as population, environment, and economy, the predicted results of this study may deviate from the actual situation, and the BAPC model needs further improvement.

## Conclusion

In summary, from 1990 to 2021, remarkable progress has been achieved in global trachoma prevention and control, with disease burden declining across all SDI regions. Notably, China, as the most populous country in the world, successfully eliminated blinding trachoma. This achievement validates the effectiveness of the World Health Organization’s SAFE strategy—Surgery, Antibiotics, Facial cleanliness, and Environmental improvement. Furthermore, China’s unique governance model has provided valuable experience for global trachoma elimination efforts. Through government-led, multi-sectoral collaboration, trachoma control was integrated into national development plans. Medical institutions, communities, and schools were mobilized to form an extensive screening and treatment network covering both urban and rural areas, significantly reducing prevalence in rural regions. By combining international standards with localized approaches, China innovatively proposed a phased prevention and control strategy based on the WHO SAFE framework: focusing on transmission interruption in the early stages, followed by enhanced surveillance and targeted interventions in later stages. Although blinding trachoma has been eliminated in China, elderly individuals over 60 years of age still require long-term follow-up due to complications from past infections, such as trichiasis and corneal opacity. Additionally, trachoma burden demonstrates notable disparities by gender and age, indicating that women and the elderly should be prioritized in future control efforts. To effectively reduce the social and healthcare burden caused by trachoma, it is essential to implement targeted screening among high-risk populations, following the principles of case detection, diagnosis, and treatment. At the same time, public health education should be strengthened for the general population to further mitigate the disease burden of trachoma in China.

## Supporting information

S1 Data The data used and analyzed during the study was available as supporting information. (XLSX)

S2 Data Trachoma data from various regions of the world in 1990 and 2021. (XLSX)
